# Supplementary material for: Identification of photoperiod-induced specific miRNAs in the adrenal glands of Sunite sheep (Ovis aries)
Source: Front Vet Sci. 2022 Jul 22;9:888207. doi: 10.3389/fvets.2022.888207 (PMC9354845; doi:10.3389/fvets.2022.888207)
Supplement: Supplementary file 1 [file Data_Sheet_1.ZIP › Supplementary file/miRDeep2 file.zip!/miRDeep2 file/Novel_Structure/LP42A1/Novel_414.pdf]

Provisional ID	:	NC_019459.2_4732
Score total	:	2
Score for star read(s)	:	-1.3
Score for read counts	:	0
Score for mfe	:	1.7
Score for randfold	:	1.6
Score for cons. seed	:	
Total read count	:	88
Mature read count	:	54
Loop read count	:	0
Star read count	:	34

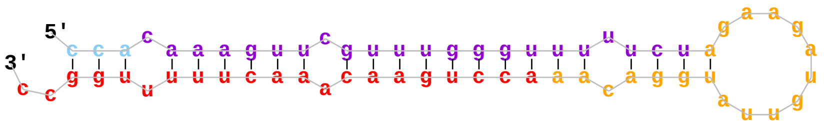

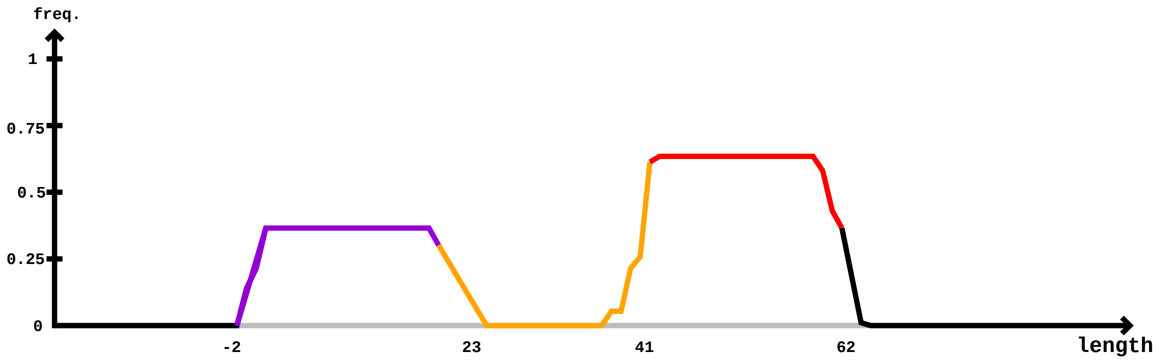


```
                        Star                                     Mature
5'- auaugguauuaaauuggccacaaaguucguuuggguuuuucuagaagauguuauggacaaaccugaacaaacuuuuuggccagcccaauaccauuaggugccaucuacua   -3'   obs
    auaugguauuaaauuggccacaaaguucguuuggguuuuucuagaagauguuauggacaaaccugaacaaacuuuuuggccagcccaauaccauuaggugccaucuacua         exp
    ..((((((((...(((((((.((((((.((((((((((.((((..........)))).)))))))))).)))))).)))))))...))))))))(((((...)))))...   reads   mm   sample
    ..................caAaaaguucguuuggguuuu.......................................................................   5       1    seq
    ..................caAaaaguucguuuggguuuuu......................................................................   7       1    seq
    ..................caAaaaguucguuuggguuuuuc.....................................................................   1       1    seq
    ...................aAaaaguucguuuggguuu........................................................................   6       1    seq
    ...................acaaaguuUguuuggguuuuuc.....................................................................   1       1    seq
    ....................caaaguucguuuggguuuuu......................................................................   2       0    seq
    ....................Gaaaguucguuuggguuuuuc.....................................................................   1       1    seq
    ....................Aaaaguucguuuggguuuuucu....................................................................   11      1    seq
    ........................................................aAaaaccugaacaaacuuuuug................................   5       1    seq
    ..........................................................aaaUcugaacaaacuuuuugg...............................   14      1    seq
    ..........................................................aaaAcugaacaaacuuuuuggc..............................   1       1    seq
    ...........................................................aaUcugaacaaacuuuuuggc..............................   4       1    seq
    ............................................................aUcugaacaaacuuuuuggcc.............................   25      1    seq
    ............................................................aUcugaacaaacuuuuuggcca............................   8       1    seq
    .............................................................Ucugaacaaacuuuuuggc..............................   1       1    seq
    .............................................................Ucugaacaaacuuuuuggccag...........................   1       1    seq
```
